# Psychotic spectrum features in borderline and bipolar disorders within the scope of the DSM-5 section III personality traits: a case control study

**DOI:** 10.1186/s40479-022-00205-w

**Published:** 2023-01-16

**Authors:** Joana Henriques-Calado, Rute Pires, Marco Paulino, João Gama Marques, Bruno Gonçalves

**Affiliations:** 1 Faculdade de Psicologia, Universidade de Lisboa, Alameda da Universidade, 1649-013 Lisboa, Portugal; 2 CICPSI, Faculdade de Psicologia, Universidade de Lisboa, Alameda da Universidade, 1649-013 Lisboa, Portugal; 3 Clínica Universitária de Psiquiatra e Psicologia Médica, Faculdade de Medicina, Universidade de Lisboa, Avenida Professor Egas Moniz, 1649-028 Lisboa, Portugal; 4 Consulta de Esquizofrenia Resistente, Hospital Júlio de Matos, Centro Hospitalar Psiquiátrico de Lisboa, Avenida do Brasil, 53, 1749-002 Lisboa, Portugal

**Keywords:** Borderline personality disorder, Bipolar disorder, Psychotic, Personality, Psychopathology, Personality inventory, DSM-5

## Abstract

**Background:**

Psychotic spectrum features in borderline personality disorder (PD) are a long-standing phenomenon, but remarkably, to date, they have not been the focus of many empirical studies. Moreover, the comparative studies that acknowledge their links to affective psychoses are even more scarce. Likewise, the contributions of empirical research on the DSM-5 dimensional approach to this topic are also uncommon. This study seeks to identify the best set of pathological personality traits and/or symptoms that are predictors of psychotic features (psychoticism and ideation paranoid symptoms) in borderline PD and in bipolar disorder, based on the framework of the DSM-5 section III personality traits.

**Methods:**

A cross-sectional study of two clinical samples: 1) Borderline PD group of 63 participants; 2) Bipolar disorder group of 65 participants. Self-reported assessment: Personality Inventory for DSM-5 (PID-5); Brief Symptom Inventory (BSI). A series of linear and logistic regression analyses were computed.

**Results:**

Overall, the data emerging as common predictors are detachment, negative affectivity, psychoticism, depressivity, grandiosity, suspiciousness and interpersonal sensitivity symptoms. Borderline PD has the highest score in BSI paranoid ideation which emerges as its discriminating trait (Nagelkerke *R*^2^ = .58): cognitive and perceptual dysregulation (OR: 13.02), restricted affectivity (OR: 12.09), withdrawal (OR: 11.70), anhedonia (OR: 10.98) and emotional lability (OR: 6.69).

**Conclusions:**

Besides the commonality that appears to overlap both disorders with a psychosis superspectrum, the patterns of the pathological personality-symptoms underlying the psychotic features appear to reinforce a position between schizophrenia and bipolar disorders that borderline PD may occupy, highlighting the possibility of its intersection with schizoaffective/psychosis spectra. The pathological personality nature of the psychotic features emerges as a potential comprehensive trait of the phenomenological dimensions.

## Background

The innermost relationship of the borderline concept and psychosis has been historically intertwined and can be traced back to the twentieth century [1]. Psychotic spectrum features in borderline personality disorder (PD) are a long-standing phenomenon, but remarkably, to date, they have not been the focus of many empirical studies. Moreover, the comparative studies that acknowledge their links to affective psychoses are even more scarce [1–6]. Likewise, the contributions of empirical research on the DSM-5 dimensional approach [7, 8] to this topic are also uncommon.

Returning to the influential review of Gunderson and Singer [9] in defining borderline patients, based on the main publications of psychiatric and psychoanalytic contributions, two characteristics have already been highlighted - brief psychotic experiences and the psychological testing performance with bizarre, dereistic, illogical or primitive responses - among the six features most often described as characterizing borderline conditions. The evidence reports that around 20–50% of patients with borderline PD experience psychotic symptoms [4], also that psychotic disorders are observed in 38% of these patients and the prevalence of 20% of psychotic disorder diagnosis not otherwise specified is the most common subtype [10]. In turn, other empirical studies point to approximately 75% of borderline cases experiencing transient dissociative and paranoid symptoms [5, 11]. More contemporary reviews have further emphasized that the most common symptoms are auditory hallucinations and paranoid delusions in borderline PD [2]. Although some studies [12] have noted that the psychotic symptoms seen in borderline patients are transient (quasi–or pseudohallucinations), other research [13] has drawn attention to the fact that psychotic symptoms in borderline PD patients, may not predict the development of a psychotic disorder but are often permanent and severe and call for careful consideration on the part of clinicians. Furthermore, the co-occurrence of borderline PD and psychotic symptoms is a marker of severe psychopathology and a poor outcome risk [3]. The presence of persistent psychotic symptoms in borderline PD has been attributed to their co-occurrence with other psychiatric disorders, such as mood disorders, post-traumatic stress disorder and substance use disorders [2]. Although chronic psychotic symptoms are typically associated with the schizophrenia spectrum and bipolar disorders [14], it is still difficult to distinguish psychotic-related phenomena in borderline PD from the corresponding experiences in psychotic disorders and schizophrenia, despite numerous attempts to do so [2]. Recently, state-of-the-art research has shown that in borderline PD, the psychotic symptoms in general, and the auditory verbal hallucinations in particular, display more similarities than differences to those symptoms in psychotic disorders [3, 15]. However, psychotic features in borderline PD appear to be significantly related to the context (usually stressful events) and emerge or intensify in response to situational crises [2].

Indeed, borderline PD and bipolar disorder are significant public health problems and, clinically, it has been frequently noted that distinguishing them from each other is challenging and a common diagnostic dilemma, due to their symptomatic overlap [5, 6, 16–20]. As mentioned by Marneros et al. [21], since accepting mood-incongruent symptoms as belonging to mood disorders as well as beyond schizophrenia, the risk of confusing diagnostic entities, such as “pure” mood disorders with schizoaffective disorders and to some extent with schizophrenia and schizophreniform disorders also increases. As a historical intersection, Stone [22] first reported that borderline patients often came from families with manic-depressive members and thus shifted borderline personality from a subschizophrenic to a subaffective disorder. In parallel, having observed the frequent association with recurrent mood disorders, coupled with family bipolarity and spontaneous and pharmacological excursions into brief periods of elation, Akiskal [23] placed the pathology of borderline patients in the bipolar realm. Differentiating bipolar disorder I from borderline PD is usually more straightforward due to the fact that bipolar disorder I is typically more severe and psychotic features during mania are frequently present [5, 24]. In bipolar disorder II, on the other hand, hypomanic episodes lack psychotic features and disorders are frequently incorrectly diagnosed as borderline PD due to shared features including impulsivity and emotional dysregulation [5, 24]. In an attempt to provide clarification, Benvenuti et al. [25] found that features of bipolarity were associated with psychotic experiences in borderline disorder and, also in the same vein, Perugi et al. [26] reported that borderline PD depressive patients frequently displayed a number of clinical variables classically associated with bipolarity, e.g., psychotic symptoms, mixed features and atypical features. Nevertheless, according to the literature reviews, the presence of certain borderline PD features (e.g., micropsychotic symptoms and interpersonal difficulties) with no clearly explainable link to mood fluctuations thereby challenges the condition being viewed as bipolar [17]. When dealing with this controversial and challenging subject, some authors also propose a schizophrenia spectrum psychopathology in borderline PD [27], or an approach to a schizoaffective disorder spectrum [28].

In light of the empirical evidence supporting the DSM-5 dimensional model of personality disorders [7, 8], few studies have examined maladaptive personality traits and psychosis conditions. Upon systematization of the data in the literature, it has become clear that psychotic symptomatology, schizophrenia spectrum disorders or higher risk for psychosis are linked to the psychoticism [14, 29], negative affectivity and detachment [30] domains which, in turn, are linked to the trait facets of unusual beliefs and experiences [14, 29], cognitive and perceptual dysregulation [14], suspiciousness [14] and distractibility [29]. Research has also pointed to a consideration of cognitive and perceptual dysregulation and suspiciousness traits for inclusion as the ninth borderline PD symptom criterion (i.e., stress-induced paranoia or dissociation) [31, 32]. Recently, Kotov et al. [33] proposed a psychosis superspectrum, stating that the thought disorder spectrum is composed of symptoms and maladaptive traits that range from normal reality testing to maladaptive trait psychoticism, to hallucinations and delusions. Some theories on the relationship between personality and psychotic disorders have hypothesized a latent discontinuity, with the risk of psychosis limited to a qualitatively distinct subgroup, however further research is needed in this regard [33].

Through self-report assessment, this paper seeks to identify the best set of pathological personality traits and/or symptoms that are predictors of psychotic features (psychoticism and ideation paranoid symptoms) in borderline PD and bipolar disorder, based on the framework of the DSM-5 pathological personality traits. To our knowledge, this is the first analysis of the DSM-5 dimensional model criterion B maladaptive traits to focus on the predictors of psychotic spectrum features associated with personality-psychopathology data in a simultaneous self-report of these disorders.

## Method

### Participants

This cross-sectional study consisted of a total of 128 patients distributed across two clinical samples: 1) Borderline PD sample of 63 participants aged between 18 and 64 years (*M*_age_ = 40.32 years, *SD* = 11.18), predominantly male (55.6%), with an average of 10 years of schooling; 2) Bipolar disorder sample of 65 participants aged between 19 and 76 years (*M*_age_ = 46.49 years, *SD* = 12.84), predominantly female (60%), with an average of 11 years of schooling.

Regarding the sociodemographic features, significant differences were observed in age between the borderline PD and bipolar disorder groups (*t*(126) = − 2.90, *p* = .004). No between-group differences were found in other sociodemographic variables such as sex or schooling.

Overall, the participants were of Portuguese nationality (94.5%), mostly single (54.3%), married/cohabiting (21.3%) or divorced (21.3%), while most were unemployed (50%), and lived predominantly in an urban environment (80.2%).

### Measures

*Socio-demographic questionnaire (e.g., age, sex, schooling)*

*Personality inventory for DSM-5 (PID-5)*

The PID-5 [8, 34, 35] is a self-report measure, which operationalizes the Criterion B of the dimensional personality pathology model proposed in the DSM-5 Section III. It is composed of 220 items, rated on a 4-point Likert scale ranging from 0 (very false or often false) to 3 (very true or often true), which characterize 25 empirically derived lower-level traits (facets) grouped into five higher-order trait domains of maladaptive personality variation. In our study, Cronbach’s alphas (α) for the domains had a value of .86 (Negative Affectivity), .83 (Detachment), .89 (Antagonism), .88 (Disinhibition), and .94 (Psychoticism).

*Brief symptom inventory (BSI)*

The BSI [36, 37] identifies self-reported clinically relevant psychological symptoms. The BSI consists of 53 items covering nine symptom dimensions scales. Participants rank each feeling item on a 5-point Likert scale ranging from 0 (not at all) to 4 (extremely). Rankings characterize the intensity of distress during the past seven days. In our study, Cronbach’s alphas (α) for the domains had a value of .89 (Somatization), .79 (Obsession-Compulsion), .87 (Interpersonal Sensitivity), .90 (Depression), .88 (Anxiety), .87 (Hostility), .83 (Phobic anxiety), .80 (Paranoid ideation), and .77 (Psychoticism).

### Procedure

The present study received approval and authorization from the Ethics Committee of the researchers’ affiliation institution and by the host institutions involved. The study was conducted in accordance with the latest version of the Declaration of Helsinki and in compliance with the European General Data Protection Regulation.

After the aims and procedures had been fully explained to the participants, their informed written consent was obtained containing both their and the researchers’ signatures. None of the participants received a reward for their contribution.

The samples were collected in Portuguese mental health units. In each affiliated mental health institution, there was a clinician who coordinated the sampling procedures, who selected the participants with the respective diagnoses contained in our study from the clinical databases of their institution, or from whom they were referred. In general, our collected clinical samples relied on the direct clinical evaluation of several psychiatrists, whose diagnosis had been previously discussed and agreed upon by a clinical team. It should be noted that each diagnosis is the result of a medical psychiatric evaluation, archived on the clinical records, conducted by at least three different clinicians: the assistant psychiatrist; the coding doctor, responsible for the respective Diagnosis-Related Groups (DRG) [38]; and the collaborating researcher. The codifying doctor of the hospital(s) based on WHO’s ICD-9 criteria, make diagnosis after reading all clinical records by other clinicians, either old paper (hardware) or new electronic (software) archives of each patient and, subsequently, re-encodes according to the DSM-5. Patients were selected according to their DSM-5 diagnosis (Sections I, II) and the study’s inclusion and exclusion criteria. It is emphasized that patients’ samples are exclusively based on the psychiatric clinical diagnostic method ant the comorbidity of diagnoses was eliminated at baseline. The study inclusion criteria were aged 18 years or above and the diagnostic criteria for borderline PD and bipolar disorder. The exclusion criteria for this study were intellectual disability, schizophrenia, and neurocognitive disorders. Patients with comorbid diagnoses of borderline PD and bipolar disorder were also excluded. Some patients answered the research protocol during their brief term hospitalizations, others were outpatients, admitted sequentially in the sample whenever they had a follow-up consultation. It is estimated that 25% is related to invalid protocols, dropouts and refusals to participate in the research.

#### Data analysis

The statistical analyses were conducted with the PASW Statistics Software (v. 24, SPSS Inc., Chicago, IL). Effects for *p-values* ≤ .05 were considered statistically significant.

The main objective was explored by means of the following: 1) For the purpose of description, a series of Pearson correlations and one-way Analysis of Variance (ANOVAs) were computed across the diagnostic groups (borderline PD and bipolar disorder) and scores on the PID-5 domains/facets and the BSI scales. The assumptions of this statistical method were validated by checking the normality and the homogeneity of variances. The adjusted alpha level with the Bonferroni correction procedure were used for adjustment of multiple comparisons; 2) To test the models that predict BSI’s psychoticism and paranoid ideation scales, for borderline PD and bipolar disorder groups respectively, twelve multiple linear regressions were performed using a stepwise method: model 1 - the PID-5 higher-order domains were entered as predictors; model 2 - the PID-5 lower-order trait facets were entered as predictors; model 3 - the PID-5 facets and BSI scales were entered as predictors. The sociodemographic variables (sex, age, schooling) were controlled. Collinearity diagnostics were analyzed using two indicators, VIF (Variance Inflation Factor) and Condition index values. The *R*^2^ was used to measure the global predictive capacity of the model; 3) To determine whether the PID-5 facets were able to classify the diagnostic status of borderline PD vs. bipolar disorder through the higher BSI paranoid ideation symptoms scale (*M* ≥ 1.69), as an exploration of the results observed in objective 1) - a binary logistic regression was performed using a backward wald method: the PID-5 facets were entered as predictors. Odds ratios (ORs) were obtained for each parameter, and the Nagelkerke *R*^2^ was used to measure the global predictive capacity of the model. The Hosmer–Lemeshow goodness-of-fit test (*X*^2^_HL_) was used to determine the suitability of the logistic regression model’s fit.

## Results

Table 1 reports the means, standard deviations, analysis of variance and Pearson significant correlations between all variables in both groups under study.

**Table 1:**
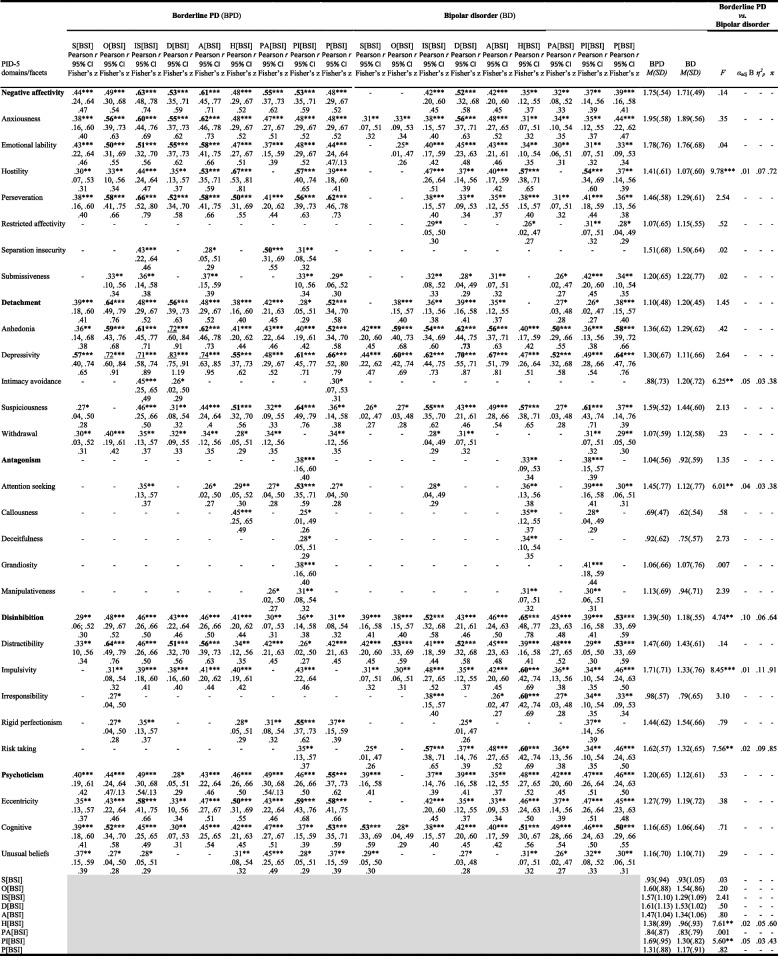
Summary of Correlations, Descriptive Statistics and Results of the Analysis of Variance on the Effect of the Clinical Groups on the Variables in Study

*Note*. * *p* < .05. ** *p* < .01. *** *p* < .001

Coefficient of determination (*r*²) of moderate and large effect sizes values correlations are in bold and underlined, respectively. Very small; small; moderate; large: 10< *r*^2^≤ .25; .25< *r*^2^≤ .50; *r*^2^> .50 (Cohen, 1988). *α*_adj_ B = the adjusted alpha level with the Bonferroni procedure; *η*^2^_*p*_ (effect size): ≤ .05 (small); ] .05; .25] (medium); ] .25; .50] (high); > .50 (very high); *π* (test power): ≥ .80; 1:00] (Cohen, 1988)

[S[BSI]: Somatization; O[BSI]: Obsession-compulsion; IS[BSI]: Interpersonal sensitivity; D[BSI]: Depression; A[BSI]: Anxiety; H[BSI]: Hostility; PA[BSI]: Phobic anxiety; PI[BSI]: Paranoid ideation; P[BSI]: Psychoticism]

**Fig. 1:**
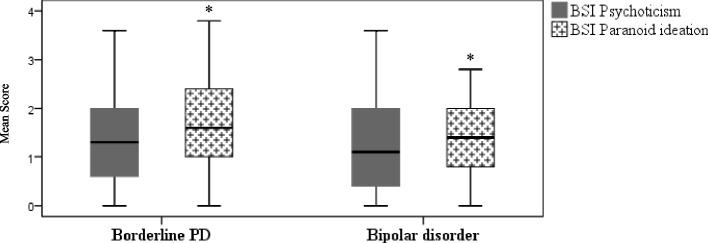
Boxplots of BSI’s psychoticism and paranoid ideation symptom scale scores for borderline PD and bipolar disorder groups. Between-group differences were observed on the BSI paranoid ideation scale (*t*(116) = 2.37, *p* = .02) (borderline PD *M* = 1.69, *SD* = .95; bipolar disorder *M* = 1.30, *SD* = .82), while no between-group differences are reported on the BSI psychoticism scale (*t*(126) = .73, *p* = .47) (borderline PD *M* = 1.20, *SD* = .65; bipolar disorder *M* = 1.12, *SD* = .61)

Figure 1 shows between-groups score differences on the BSI paranoid ideation scale, with the borderline PD group having the highest mean score.

**Fig. 2:**
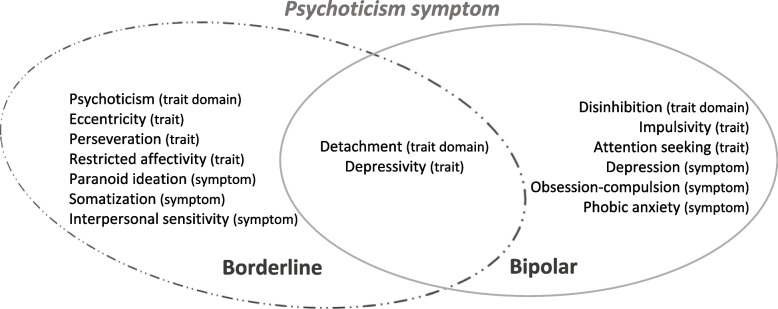
Illustration and summary of the best predictors of the BSI psychoticism symptom scale in borderline PD and bipolar disorders, based on the framework of the DSM-5 personality traits and psychopathological symptoms, regarding the specific features and similarities between these disorders

**Fig. 3:**
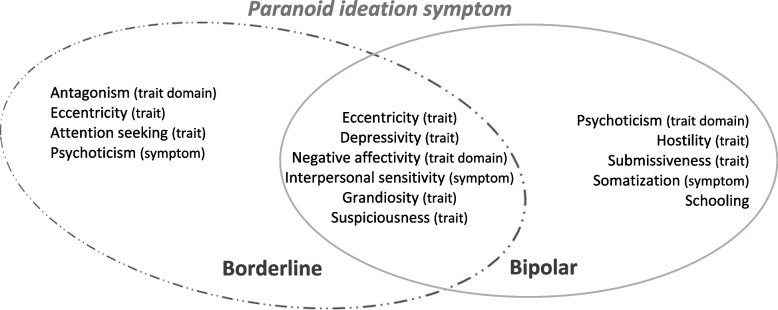
Illustration and summary of the best predictors of the BSI paranoid ideation symptom scale in borderline PD and bipolar disorders, based on the framework of the DSM-5 personality traits and psychopathological symptoms, regarding the specific features and similarities between these disorders

Table 2 reports the results obtained by multiple linear regression models for the prediction of the BSI’s psychoticism and paranoid ideation scales as criterion variables, in the clinical groups under analysis. Overall, the DSM-5 personality pathological traits (PID-5) and psychopathological symptoms (BSI scales), which are predictors of psychotic symptom features (BSI’s psychoticism and paranoid ideation), in borderline PD and bipolar disorder groups are illustrated and summarized in Figs. 2 and 3.

**Table 2:**
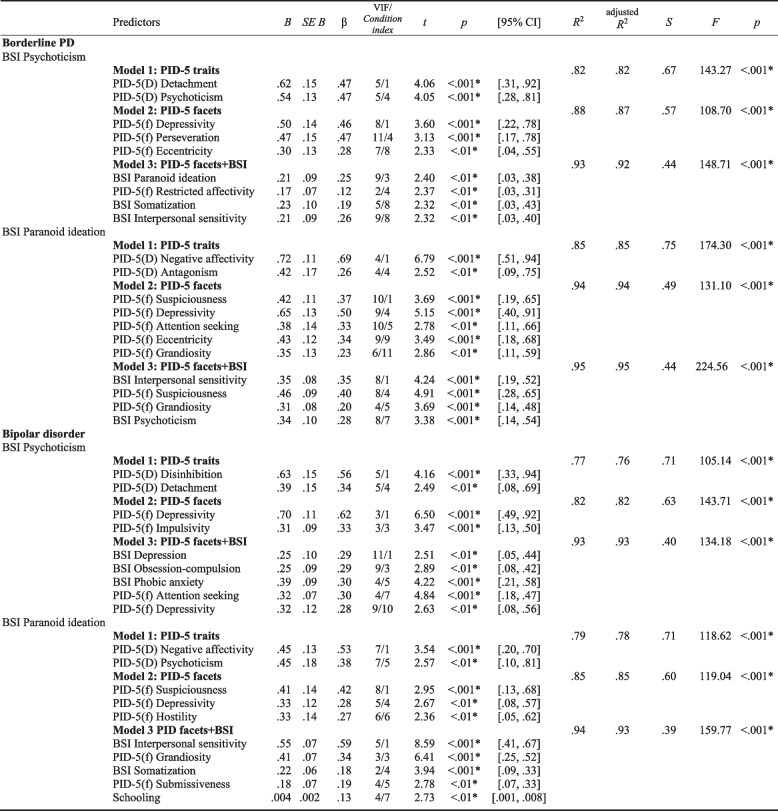
Summary of Predictive Models of BSI’s Psychoticism and Paranoid Ideation Symptom Scales for Borderline PD and Bipolar Disorder

*Note*. * Two-tailed

Collinearity statistics: VIF [Variance Inflation Factor] ≤ 10 indicative of inconsequential collinearity (Myers, 1986); Condition index: ≤ 10 indicate weak dependencies among the independent variables, 30-100 indicate moderate to strong dependencies, > 100 indicate serious multicollinearity (Belsley et al., 1980; Rawlings et al., 1998); S [Standard Error of the Regression]

Table 3 presents the results of the logistic regression analysis and shows the significant coefficients of the PID-5 personality traits in the prediction of borderline PD vs. bipolar disorder through the higher mean score of BSI paranoid ideation symptoms. The regression model was significant (χ^2^ = 27.66, *df* = 9, *p* < .001, − 2 Log likelihood = 40.27), accounting for 58% of the variance (Nagelkerke’s *R*^2^ = .58; Cox & Snell *R*^2^ = .43), the Hosmer–Lemeshow test: *X*^2^_HL_(8) = 7.21, *p* = .514, suggests that the model is a good fit. The obtained model showed that 5 variables uniquely and significantly contributed to the model associated with higher odds of outcome (OR > 1), thus emerging as differential trait predictors of borderline PD.

**Table 3:** Summary of Logistic Regression Model Predicting Borderline PD vs. Bipolar Disorder through Higher Mean Score of BSI Paranoid Ideation Symptom Scale

	B (S.E.)	Wald (χ^2^)	*p*	OR [95% CI]	Nagelkerke *R*^2^
**Model**					.58
PID-5(f) Withdrawal	2.46 (1.06)	5.46	<.01*	11.70 [1.48, 92.68]	
PID-5(f) Restricted affectivity	2.49 (1.03)	5.86	<.01*	12.09 [1.61, 90.84]	
PID-5(f) Anhedonia	2.40 (1.32)	3.29	<.05*	10.98 [.83, 146.00]	
PID-5(f) Unusual beliefs & experiences	−2.32 (1.14)	4.12	<.05*	.10 [.01, .92]	
PID-5(f) Depressivity	−6.69 (2.27)	8.64	<.001*	.001 [.001, .11]	
PID-5(f) Cognitive & perceptual dysreg.	2.57 (1.26)	4.13	<.05*	13.02 [1.10, 154.6]	
PID-5(f) Emotional lability	1.90 (1.01)	3.57	<.05*	6.69 [.93, 48.06]	
PID-5(f) Attention seeking	−3.67 (1.25)	8.65	<.001*	.03 [.002, .29]	

*Note*. * Two-tailed; *B* logistic regression coefficient. *S.E*. standard error of the logistic regression coefficient. *OR* odds ratio. PID-5 (f) PID-5 facet

## Discussion

### General and common main findings of borderline PD and bipolar disorders

The main set of data emerging from this study as common predictors are the pathological personality traits of detachment, negative affectivity, psychoticism, depressivity, grandiosity, suspiciousness, and interpersonal sensitivity symptoms (Table 2; Figs. 2 and 3). The first general finding is that depressivity (the lower trait-based detachment domain) emerges as a common pathological personality trait marker predictor of psychotic phenomena symptomatology (psychoticism and paranoid ideation symptoms) in both borderline PD and bipolar disorders. Thus, there appears to be a commonality of these data with the proposed psychosis superspectrum framework [33], where thought disorder and detachment spectra are superimposed. In fact, the interpersonal domain of detachment, an internalizing psychopathological tendency [39], is also highlighted, and has been shown to have a closer relationship with the psychotic or thought disorder spectrum [40] in prior studies. In fact, on another level of analysis, in conjunction with psychoticism, it may also be conceptualized as a sub-dimension of the internalizing spectrum of disorders [41]. Additionally, other published studies found that bipolar disorder/mania symptoms loaded on either the psychosis or internalizing dimensions, with borderline PD and major depressive disorders equally fitting in this latter dimension, reflecting an affective dysregulation foundation [33, 40–44]. It can be argued that these data also feature psychoticism as a core trait domain posits as a general factor of personality disorder [45–47], or as a core personality dysfunction [48]. In this follow-up, there is evidence that psychoticism can capture clinically relevant aspects of severe mental illness associated with psychosis and other related characteristics, indicating possibly shared patterns of personality expression [14, 49], demonstrating a degree of non-specificity in the patterns across symptoms of personality disorders and non-personality disorders, similarly to some other evidence in the literature [49].

On further examination of these data, and focusing specifically on the psychoticism symptom, the latter appears to be related to the common DSM-5 personality predictors of detachment and depressivity in both pathologies of our research (Table 2; Fig. 2). It should be noted that the DSM-5 includes depression as a dimension of psychosis and, in turn, depression trait may be an important contributor to the clinical heterogeneity of schizophrenia [50]. Delusional and hallucinatory experiences are known to occur in both manic and depressive conditions [51]. Furthermore, findings suggest that a co-occurring genetic vulnerability for both depressive and psychotic symptomatology exists at both a clinical and subclinical level [52]. Clinically, this is reflected in diagnoses such as schizoaffective or mood disorders with psychotic features, in which depressive and psychotic symptoms co-occur [52]. It is possible that trait depression reflects chronically heightened affective reactivity and may influence the development of psychotic-like experiences via the affective pathway [50, 53, 54].

In turn, considering the paranoid ideation symptom in particular (Table 2; Fig. 3), in this study, from a general point of view, it appears to be related to several common DSM-5 personality predictors: the negative affectivity domain, grandiosity trait-based antagonism, depressivity and suspiciousness trait-based detachment, and interpersonal sensitivity symptoms. Taken together, these data may suggest the presence of an affective paranoia complex as a common background to both disorders. Recent studies have shown that the paranoia continuum has links with negative affectivity, depressed mood, stress and interpersonal sensitivity, mistrust and ideas of reference [55–58]. The paranoia mechanism is highlighted as an underlying co-occurrence of altered mood states and psychosis, reflecting the presence of difficulties in interpersonal relationships [58]. Interestingly, our data also reflect Kraepelin’s position towards the aetiology of paranoia on the one hand, understanding it more as personality development rather than a disease [59] and, on the other hand, more contemporary positions suggesting that emotional dysregulation plays a mediating role in hallucinations and paranoia [60].

### Specific and differential main findings for borderline PD and bipolar disorders

A further finding stemming from our analyses involves other features associated with the psychotic phenomenon (psychoticism and paranoid ideation symptoms) that emerge as being specific to borderline PD and bipolar disorder. Overall, the specific traits of antagonism, eccentricity, perseveration, restricted affectivity, and symptoms of psychoticism and paranoid ideation are highlighted in borderline PD; and in turn, in bipolar disorder, the traits of disinhibition, impulsivity, hostility, submissiveness, and symptoms of depression, obsession-compulsion and phobic anxiety are shown as specific predictors (Table 2; Figs. 2 and 3).

It is noteworthy that the predictive models observed present a set of personality traits with and without associated symptoms that point to a differential pattern in the prediction of each psychotic feature under study and in each of the different disorders. In borderline PD, the interpretations of two dimensions may be advanced: 1) the schizoaffective/psychosis [33] dimension [related to the psychoticism symptom dimension] (detachment, psychoticism, depressivity, perseveration, eccentricity, restricted affectivity, and symptoms of somatization, interpersonal sensitivity and paranoid ideation) and; 2) the internalizing/antagonistic [61, 62] dimension [related to the paranoid ideation symptom] (negative affectivity, antagonism, depressivity, suspiciousness, eccentricity, attention seeking, grandiosity, and symptoms of interpersonal sensitivity and psychoticism). In bipolar disorder, the following interpretations are proposed: 1) the internalizing/disinhibition [61, 62] dimension [related to the psychoticism symptom] (disinhibition, detachment, depressivity, attention seeking, impulsivity and symptoms of phobic anxiety, depression, obsession-compulsion) and; 2) the emotional dysfunction (internalizing and somatoform spectra)/psychosis [33, 61] dimension [related to the paranoid ideation symptom] (negative affectivity, psychoticism, suspiciousness, grandiosity, depressivity, hostility, submissiveness, and symptoms of interpersonal sensitivity and somatization).

In this regard, some of our results are consistent with other studies suggesting that borderline PD loads onto the internalizing spectrum, while its association with externalization is via antagonism [61, 62], demonstrating a connection to unstable negative affect [63]. The antagonistic spectrum is also associated with paranoid PD, and negative affectivity and low effortful control predict borderline PD, representing a consistent constellation of temperamental traits that acts as an antecedent to the externalizing superspectrum [61, 62]. On the other hand, several studies have found that indicators of mania/bipolar disorder fall within the internalizing spectrum and often help to define its distress subfactor and have also important connections to thought disorder spectrum-psychosis [33, 61]. As far as the disinhibition identified in our study regarding bipolar disorder is concerned, it is likely to be associated with mania/hypomania [64, 65]. A hypothetical link between the schizoaffective-psychosis [33, 66] dimension and borderline PD and, in turn, the emotional dysfunction-psychosis [33, 61, 67] dimension and bipolar disorder, appears to arise in this study underpinning the psychotic spectrum features. This appears to reinforce the distinction between borderline PD and bipolar disorder, adding evidence to the literature review that underlines that these two conditions are different and can be distinguished [68–70].

The last hallmark in our results is the evidence that borderline PD has the highest score in the BSI’s paranoid ideation (Fig. 1), which is in line with some of the findings in the literature [2, 3, 5, 11, 15, 31, 32]. Thus, the investigation of discriminant pathological personality model predictors (Nagelkerke *R*^2^ = .58) for borderline PD and bipolar disorders through higher mean values of paranoid ideation symptoms is worthy of mention (Table 3): cognitive and perceptual dysregulation (OR: 13.02), restricted affectivity (OR: 12.09), withdrawal (OR: 11.70), anhedonia (OR: 10.98) and emotional lability (OR: 6.69). Psychoticism has been described as partially capturing features of borderline PD in terms of cognitive and perceptual dysregulation, which includes features of dissociation proneness [71]. This set of data, associated with the afore-mentioned evidence, appears to suggest a possible overlap of schizoaffective/psychosis spectra and is at the crossroads of the challenging debate that claims a borderline PD-schizophrenia-schizoaffective-bipolar spectra [16, 22, 23, 25–28, 72, 73]. It should be noted that schizoaffective disorder recognizes the diagnostic relevance of mood symptoms in psychotic patients, linked to schizophrenia (psychosis) and mood disorders, occupying an intermediate position between schizophrenia and affective disorders [66, 74–76]. In this regard, a bridge may be established with the concept recently proposed by Tyrer et al. [77], namely Galenic syndromes, which underlines the entwined relationship between personality and some mental disorders, acknowledging a broader link between personality pathology and psychopathology.

This study presents several limitations, such as the small size of the samples, the absence of data on the participants’ prior clinical history and current treatment, the fact that mood and psychotic states were not assessed, and bipolar disorder was assigned without the specification of subgroups. Additionally, the possibility of the sample being contaminated by undiagnosed pathologies such as psychosis/personality disorders of organic/toxic aetiology is a further limitation. Its cross-sectional design may also be a major limitation, as borderline and bipolar disorders are primarily characterized by a fluctuating long-term course of symptoms during the life-span, thereby possibly resulting in false-positive diagnoses. However, in this study, the comorbidity of diagnoses was eliminated, which may have facilitated the minimization of errors [69]. In addition, this design does not allow for conclusions to be drawn on temporal and causal relationships between the psychiatric diagnoses in terms of outcomes, thus constituting a potential confounder. Self-report may also be considered a potential and partial limitation as far as the psychiatric samples in this study are concerned, since although there is no consensus with regard to the DSM-5 assessment that diagnostic interviews are more valid than self-reports, the combined use of these methods is deemed optimal for assessing functional outcomes or criteria in borderline PD [78–81], hence the use of interview assessments in prospective studies is also recommended. Prudence is necessary in interpreting the relationship between the variables under study, given that some items are very similar in the two instruments used. However, this similarity also shows how difficult it is sometimes to distinguish a pathological personality trait from a symptom, following the intrinsic nature of psychopathology. Future research should focus on the efficacy of the framework of the DSM-5 section III personality traits (Criterion B) / personality impairment (Criterion A), as a potential psychotic nature discriminant of the affectivity instability-psychotic phenomenon in borderline pathology, through comparisons with the schizophrenia-affective disorders spectrum.

## Conclusions

The findings support the DSM-5 section III personality traits as differentiating model predictors of psychotic phenomena in borderline PD through bipolar disorder. Furthermore, they reinforce the joint use of symptom-related pathological functioning and a dimensional range grounded on personality traits. Besides the commonality that appears to overlap both disorders with a psychosis superspectrum, the patterns of the pathological personality-symptoms underlying the psychotic features appear to reinforce a position between schizophrenia and bipolar disorders that borderline PD may occupy, highlighting the possibility of its intersection with schizoaffective/psychosis spectra. The pathological personality nature of the psychotic features emerges as a potential comprehensive trait of the phenomenological dimensions.

## Data Availability

The datasets used and/or analysed during the current study are available from the corresponding author on reasonable request.
